# Intracapsular Enucleation of a Cervical Phrenic Nerve Schwannoma: A Rare Case Report

**DOI:** 10.1002/ccr3.71570

**Published:** 2025-12-01

**Authors:** Mahboobe Asadi, Amirhossein Ehsani, Alireza Movahedi, Maryam Parvizi

**Affiliations:** ^1^ Department of Otolaryngology and Head and Neck Surgery Shahid Beheshti University of Medical Sciences Tehran Iran; ^2^ Department of Pathology Shahid Beheshti University of Medical Sciences Tehran Iran

**Keywords:** neck mass, neck schwannoma, phrenic nerve, phrenic schwannoma

## Abstract

Phrenic nerve schwannomas in the neck are exceptionally rare and can present as asymptomatic lateral neck masses. Intraoperative nerve identification is critical for the preservation of diaphragmatic function. Surgeons should include this rare entity in the differential diagnosis of cervical schwannomas to avoid misdiagnosis and inadvertent nerve injury.

## 1 Introduction

Schwannomas can arise from any peripheral, cranial, or autonomic nerve in the neck. These Scarce neurogenic tumors usually present as an encapsulated, slow‐growing mass. In the neck region, extra cranial schwannomas are more common in parapharyngeal space. Among cervical schwannomas, the vague nerve is the most common site of origin [1].

Cervical phrenic nerve schwannomas are benign tumors originating from Schwann cells of the phrenic nerve that innervates the diaphragm. Although schwannomas are relatively common among peripheral nerves in the head and neck, involvement of the cervical segment of the phrenic nerve remains an unusual finding. However, schwannoma of cervical pherenic nerve is rare; a few sporadic cases are reported in the literature. Cervical Schwannoma with phrenic nerve involvement is usually asymptomatic, occasionally hiccups have been reported. Imaging modalities, especially computed tomography (CT) and magnetic resonance imaging (MRI) with contrast are essential for accurate localization and surgical planning [2, 3, 4, 5, 6, 7].

In this report, we present a rare case of phrenic nerve schwannoma located in the neck. Our report is in line with the SCARE 2020 criteria [8].

## 2 Case History

A 49‐year‐old woman presented with a 6‐month history of a progressively enlarging painless swelling in the right lower neck region. She reported no history of dyspnea, cough, hoarseness, and dysphagia. No history of trauma or prior surgery was noted. Physical examination revealed a firm, mobile, non‐tender mass approximately 3 cm in diameter located in the right supraclavicular region. No cervical lymphadenopathy was palpated.

## 3 Differential Diagnosis, Investigations and Treatment

**FIGURE 1:**
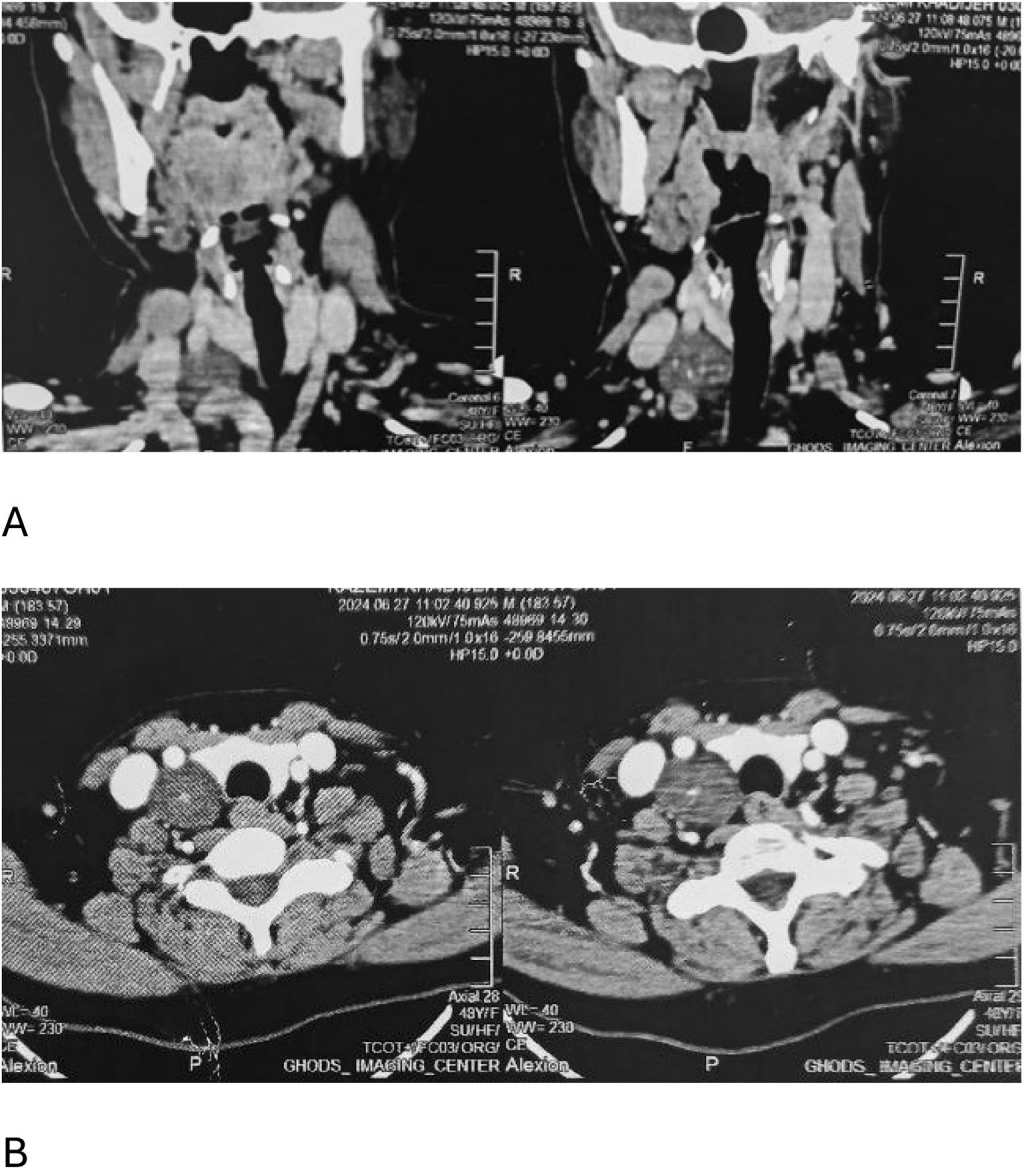
(A) Preoperative coronal CT scan with intravenous contrast showing a right‐sided hypodense and heterogenous mass (B) Preoperative axial CT scan.

Ultrasonography demonstrated a hypoechoic, well‐defined mass adjacent to the right sternocleidomastoid muscle. The patient was referred to our clinic with a neck Computed tomography (CT) scan that revealed a soft tissue well‐delineated mass in the right side of neck, measuring 5 × 4.4 × 6 cm. The solitary mass was located medial to the right jugular vein and carotid artery, with a clear plane of demarcation (Figure 1). The differential diagnosis of a solitary, painless, cervical mass encompasses various benign and malignant entities, including reactive or metastatic lymphadenopathy, neurogenic tumors, congenital mass, thyroid and muscular pathologies. Imaging modalities aid in characterizing the lesion's location, morphology, and relation to adjacent structures, while definitive diagnosis relies on cytological or histopathological analysis. Fine‐needle aspiration cytology result was compatible with a benign spindle cell tumor. The surgical excision was performed under general anesthesia. Intraoperatively, a solitary mass was found originated from the right cervical phrenic nerve. Besides, the tumor stimulation with a nerve probe at 0.5 mA was associated with diaphragm activation, which confirm the mass origin. The tumor was meticulously separated from surrounding tissues using microsurgical techniques and magnification. An intracapsular enucleation approach was employed: the tumor capsule was incised longitudinally, and the tumor was dissected free from intact nerve fascicles, minimizing trauma to the phrenic nerve. Hemostasis was secured with bipolar cautery, avoiding thermal injury. The nerve sheath was preserved whenever possible. After enucleation intraoperative stimulation of the phrenic nerve resulted in activation of the diaphragm.

## 4 Conclusion and Results (Outcome and Follow‐Up)

**FIGURE 2:**
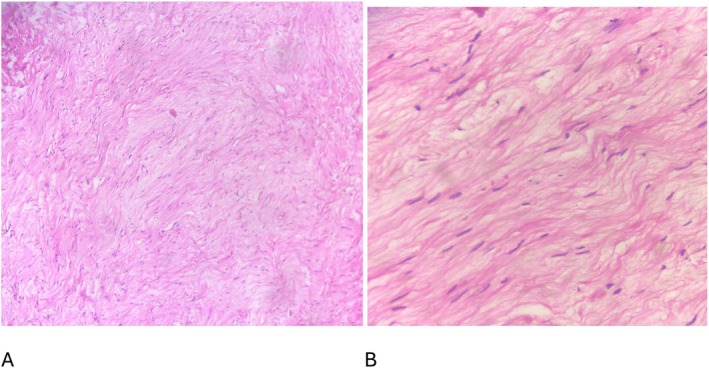
Hematoxylin and eosin (H&E) histopathology of mass. Sections show soft tissue neoplasm composed of fascicles of bland looking spindle cells with wavy nuclei. Foci of hypo and hypercellular area are present also. (A) H&E, 100× magnification, (B) H&E, 400× magnification.

**FIGURE 3:**
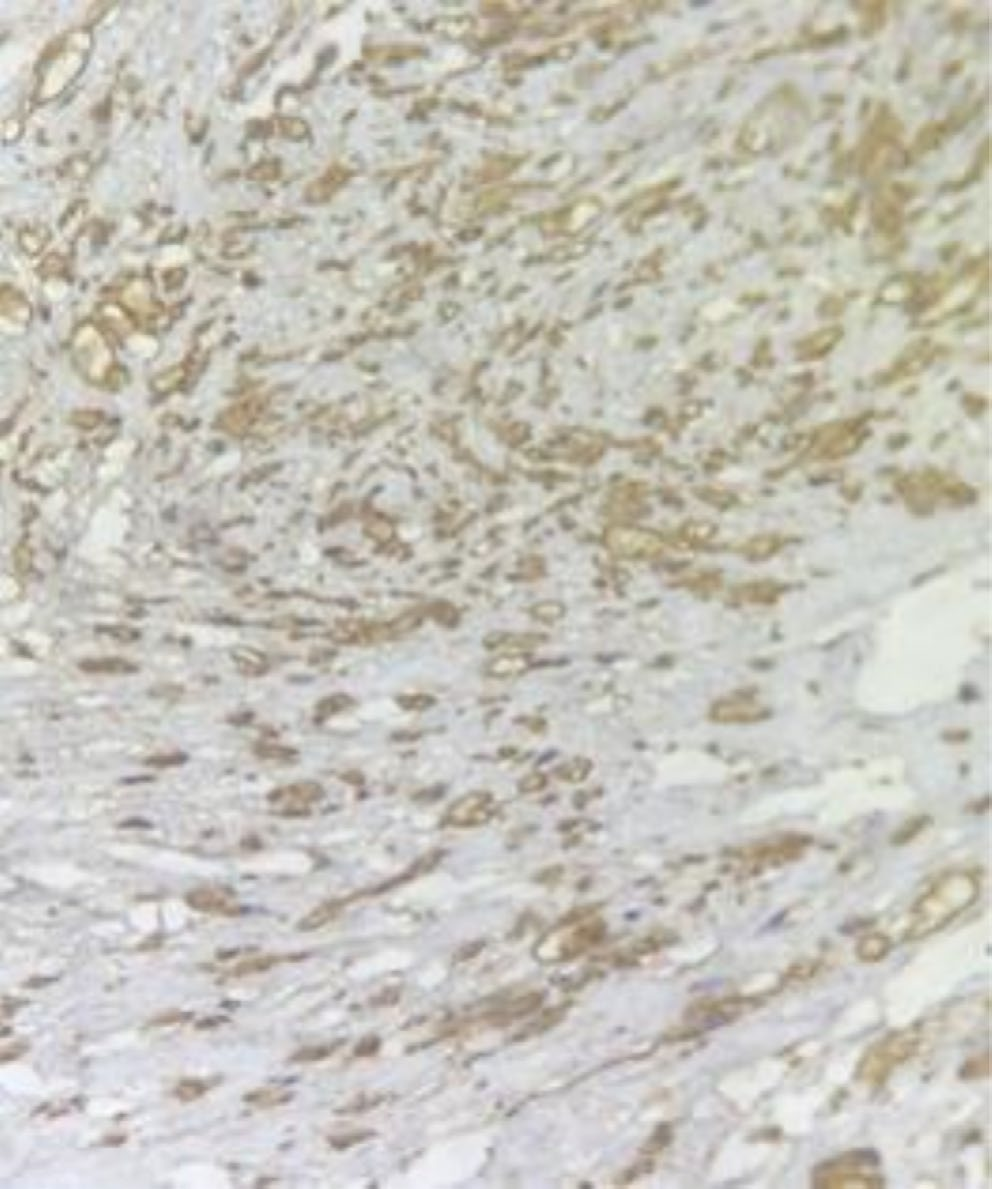
IHC study showed S100 diffuse strong positivity in tumoral cells.

The patient's postoperative course was uneventful with no new respiratory symptoms or evidence of phrenic nerve palsy. Histopathology confirmed a benign schwannoma showing typical Antoni A and Antoni B areas and strong S‐100 positivity (Figures 2 and 3). At 12 months, there was no evidence of recurrence, and diaphragmatic motion remained normal on follow‐up ultrasound.

## 5 Discussion

In this report we present an asymptomatic cervical schwannoma originated from phrenic nerve.

Schwannomas, also known as neurilemmomas, are rare, solitary, encapsulated mass with the growing rate of about 3 mm per year. Also, these tumors are predominantly benign with a malignant transformation rate of less than 2% [9].

Schwannoma arising from the cervical phrenic nerve is a rare entity. In 1988 Grunstein et al., described 16 cases of phrenic nerve schwannomas, that only one was originated from cervical part (5). 10 years later, Le Pimpec‐Bartges et al., reported 13 cases of phrenic nerve schwannoma s that all were intrathoracic [10].

According to a report by Chan et al. at the end of 2022 only 5 cases of cervical phrenic nerve schwannoma were documented in the English literature [11].

Besides, the nerve of origin is important to consider as it guides the surgeons and has a direct correlation with surgical complications. Although these tumors are predominantly asymptomatic, specific symptoms especially in mediastinal schwannoma such as dry cough, diaphragmatic paralysis, hiccups, obstruction of airways, and pneumonia were reported. Our case was referred for solitary neck mass, without symptoms of phrenic nerve involvement [2].

However, radiological imaging is indispensable for diagnosis, Ultrasound serves as a useful initial modality. MRI remains the gold standard, allowing detailed characterization of tumor size, margins, internal composition, and relation to adjacent structures. Typical MRI features include a well‐circumscribed mass with heterogeneous signal intensity on T2‐weighted images and homogeneous contrast enhancement. Interestingly, electrophysiological studies such as EMG may aid in assessing phrenic nerve integrity but may be normal if the tumor is slow‐growing and noninvasive, as in our patient [12].

Complete surgical excision remains the definitive treatment for phrenic nerve schwannomas, aiming to achieve tumor removal while preserving nerve function. Surgical challenges include the small diameter and delicate nature of the phrenic nerve, the proximity to critical neurovascular structures in the neck, and the risk of postoperative diaphragmatic paralysis [13].

Intracapsular enucleation under magnification with microsurgical instruments is the preferred technique that minimizes nerve injury. Intraoperative nerve monitoring can provide real‐time feedback on phrenic nerve function, though it is not universally available. In cases where nerve sacrifice is unavoidable, diaphragmatic plication or phrenic nerve reconstruction may be considered, but these are more invasive.

Phrenic nerve injury is the primary concern in phrenic nerve schwannoma surgery. Diaphragmatic paralysis or paresis on the affected side may manifest as dyspnea, decreased exercise tolerance, and respiratory insufficiency, especially in patients with preexisting pulmonary disease.

Postoperative diaphragmatic dysfunction can sometimes be managed with supportive respiratory therapy. Rarely, surgical interventions like diaphragmatic plication or phrenic nerve grafting may be considered [14].

Phrenic nerve schwannomas are usually benign with a very low risk of malignant transformation. Recurrence after complete excision is rare [15]. Postoperative diaphragmatic palsy can significantly affect quality of life; therefore, careful dissection and nerve preservation are critical. Our patient experienced no diaphragmatic dysfunction postoperatively, consistent with outcomes reported in similar case series [14].

## Author Contributions

**Mahboobe Asadi:** conceptualization, supervision, visualization, writing – original draft, writing – review and editing. **Amirhossein Ehsani:** investigation, visualization, writing – original draft, writing – review and editing. **Maryam Parvizi:** data curation, resources, supervision, writing – original draft, writing – review and editing. **Alireza Movahedi:** investigation, project administration, writing – original draft, writing – review and editing.

## Funding

The authors have nothing to report.

## Ethics Statement

Ethical approval for this study was approved by the Ethical Committee of Shahid Beheshti University of Medical Sciences, Tehran, Iran. The present study complies with ethical and research standards involving humans. This article does not contain any studies involving animals performed by any of the authors.

## Consent

Written informed consent was obtained from the patient for publication of this case report and the accompanying images. A copy of the written consent is available for review by the editor‐in‐chief of this journal.

## Conflicts of Interest

The authors declare no conflicts of interest.

## Data Availability

Data in the current study is available from the corresponding author on reasonable request.
